# Assisted reproductive techniques in Latin America: The Latin American Registry, 2017

**DOI:** 10.5935/1518-0557.20200029

**Published:** 2020

**Authors:** Fernando Zegers-Hochschild, Javier A. Crosby, Carolina Musri, Maria do Carmo B. de Souza, A. Gustavo Martinez, Adelino Amaral Silva, José María Mojarra, Diego Masoli, Natalia Posada

**Affiliations:** 1 Unit of Reproductive Medicine, Clínica Las Condes, Santiago, Chile; 2 Program of Ethics and Public Policies in Human Reproduction, University Diego Portales, Santiago, Chile; 3 Latin American Network of Assisted Reproduction (REDLARA), Montevideo, Uruguay; 4 Fertipraxis, Rio de Janeiro, RJ, Brazil; 5 Fertilis Medicina Reproductiva, San Isidro, Buenos Aires, Argentina; 6 Genesis Centro de Assistência em Reprodução Humana Ltda, Brasília, DF, Brazil; 7 URA, Unidad de Reproducción Asistida de Hospital CIMA Hermosillo, Hermosillo, Sonora, Mexico; 8 INSER, Medellín, Colombia

**Keywords:** Assisted reproductive technology, Latin American Registry, ART utilization, success rates, perinatal outcome

## Abstract

**Research question:**

What was the utilization, effectiveness and safety of assisted reproductive techniques performed in Latin America during 2017.

**Design:**

Retrospective collection of multinational data on ART performed in 188 institutions from 15 Latin American countries.

**Results:**

We are reporting 93,600 initiated cycles, 16,976 deliveries and the birth of 20,404 babies. ART utilization was 221 cycles/million inhabitants (15 to 535). Despite women aged ≥40 represented 30.5% of fresh IVF/ICSI, after removing freeze-all cycles, delivery rate per oocyte retrieval was 19.9% for ICSI and 20.2% for IVF. Overall, single embryo transfer (SET) represented 26.9% of fresh transfers, with 18.2% delivery rate per transfer; increasing to 32.3% in elective SET. Delivery rate in double embryo transfers (DET) was 28.3% increasing to 37.3% with elective DET. This 5% increment in births in eDET over eSET resulted in10-fold increase in twin births, almost 3 weeks’ shorter gestations and 3-fold increase in perinatal mortality. Delivery rate in frozen/thawed SET, reached 25.5% increasing to 30.8% with DET; the majority being blastocysts transfers. Of all births, 67% were singletons, 31.4% twins, and 1.6% triplets and higher. Overall, preterm deliveries reached 9.5% in singletons, 64.3% in twins and 97.9% in triplets; and perinatal mortality was 9.4‰ in singletons, 25.3‰ in twins, and 63.3‰ in high-order multiples.

**Conclusions:**

The number of initiated cycles slowly increases. Frozen embryo transfers, blastocyst transfers and SET are also increasing. Our data shows that especially in young women and oocyte recipients, when there is more than one blastocyst for transfer, elective SET should be the rule.

## INTRODUCTION

This is the 29^th^ report of the Latin American Registry of Assisted Reproduction (RLA) established in 1990 as the first multinational and regional registry of assisted reproductive techniques (ART). Since 2012, reports are published simultaneously in *Reproductive BioMedicine Online* and in *JBRA Assisted Reproduction*, the official journal of the Latin American Network of Assisted Reproduction (REDLARA). Results from previous years can be downloaded from www.redlara.com. This report provides information on utilization/availability, effectiveness, safety and perinatal outcomes of ART treatments initiated between January 1^st^ and December 31^st^, 2017, and babies born up to September 2018.

## MATERIALS AND METHODS

Data on ART were collected from 188 centers in 15 countries in Latin America (Supplementary Table 1), covering fresh autologous cycles of in vitro fertilization (IVF) and intracytoplasmic sperm injection (ICSI); Preimplantation genetic testing (PGT); frozen embryo transfer (FET); oocyte donation (OD) including the transfer of both fresh and frozen/thawed embryos; fertility preservation (FP); and frozen/warmed oocyte cycles, both autologous and heterologous (FTO).

**Table 1:** Assisted reproduction techniques reported in Latin America, 2017

Country	Centers	FP	FRESH	FET	OD	FTO	Total
**Argentina**	28	842	9,582	4,660	4,533	437	20,054
**Bolivia**	3	3	391	29	206	25	654
**Brazil**	63	2,491	20,065	12,282	3,069	1,235	39,142
**Chile**	10	278	1,778	1,059	710	163	3,988
**Colombia**	12	69	1,218	449	558	70	2,364
**Ecuador**	8	116	622	278	317	73	1,406
**Guatemala**	1	11	128	52	38	0	229
**Mexico**	38	399	7,222	3,030	4,905	233	15,789
**Nicaragua**	1	0	118	9	20	1	148
**Panama**	3	36	468	205	177	29	915
**Paraguay**	1	16	114	69	37	7	243
**Peru**	12	976	2,356	1,161	1,545	741	6,779
**Rep. Dominicana**	2	0	92	21	46	0	159
**Uruguay**	2	17	751	321	313	17	1,419
**Venezuela**	4	2	126	60	123	0	311
**Total**	188	5,256 (5.6)	45,031 (48.1)	23,685 (25.3)	16,597 (17.7)	3,031 (3.2)	93,600

FP, fertility preservation; FRESH, initiated fresh autologous IVF/ICSI cycles; FET, frozen autologous embryo transfer; OD, transfer of fresh or frozen embryos due to oocyte donation; FTO, includes embryo transfer cycles using autologous and donated vitrified-warmed oocytes.

This report includes treatments started between 1 January 2017 and 31 December 2017. Data on pregnancy and perinatal outcomes are obtained from follow-up of cohorts treated during this period.

As part of the accreditation program, all participating institutions agree to have their data registered and published by the RLA. Therefore, no other consent form was requested for the scientific disclosure of these data.

The method of collecting data in 2017 resembles previous years (Zegers-Hochschild *et al*., 2019), making results comparable. Definitions used refer to the latest publication of the International glossary on Infertility and Fertility Care (Zegers-Hochschild *et al*., 2017). When calculating clinical pregnancy or delivery rates per oocyte pick-up, cases of total embryo freezing were not included in the calculation.

Cumulative live birth rate was calculated, as described by Maheshwari *et al*. (2015) from cycles taking place between 2012 and 2017. We considered the first delivery after transfer of either fresh and/or frozen/thawed embryos obtained after a reference oocyte pick up. A personal identification number and date of birth identified each patient. The identification number is not universal in Latin America, so not all patients could be followed and it is also possible that cross border reproductive treatments could partially influence results, but the numbers should be small. Furthermore, only data provided by institutions using a consistent and reproducible ID number were used throughout the study period (2012 and 2017). For the purpose of reporting cumulative births, 166 institutions in 13 countries were included making sure that the use of an identification number remained throughout the study period (Paraguay and Nicaragua were excluded).

In order to test for the effect of age, number of embryos transferred and state of embryo development at transfer on the delivery rate per embryo transfer, logistic regression analysis was performed in both fresh and OD cycles. When appropriate, a chi-squared test was used to analyze independence of categorical variables. A *p*-value less than 0.05 was considered statistically significant.

## RESULTS

### Participation

188 centers in 15 countries reported 93,600 ART procedures performed during 2017. This represents more than 70% of centers in the region. The majority of centers were located in Brazil (n=63), followed by Mexico (n=38) and Argentina (n=28) (Table 1). In comparison with 2016, 6 centers which had stopped reporting resumed their participation; 9 centers either closed or stopped reporting, mainly in Venezuela and 13 centers were newly incorporated in 2017 contributing with more than 4,900 of the 8,126 new cycles reported in this period.

### Size of participating institutions and number of treatment cycles per technique

The 93,600 initiated cycles reported this year (9.5% more than 2016), correspond to the sum of IVF/ ICSI, PGT, FET, OD, FP and FTO. The mean number of initiated cycles by institution was 497.9, with wide variation; 12.8% performed ≤100 cycles; 33.5% between 100 and 300 cycles; 18.1% between 301 and 500 cycles; 20.7% between 501 and 1000 cycles, and 14.9% >1000 cycles. Overall, the major contributors were Brazil followed by Mexico and Argentina.

Out of 93,600 initiated cycles, 45,031 corresponded to IVF/ICSI (48.1%); 23,685 corresponded to FET (25.3%); 16,597 to OD (17.7%), 5,256 to FP (5.6%), and 3,031 cycles reported as FTO (3.2%) (Table 1).

**Figure 1:**
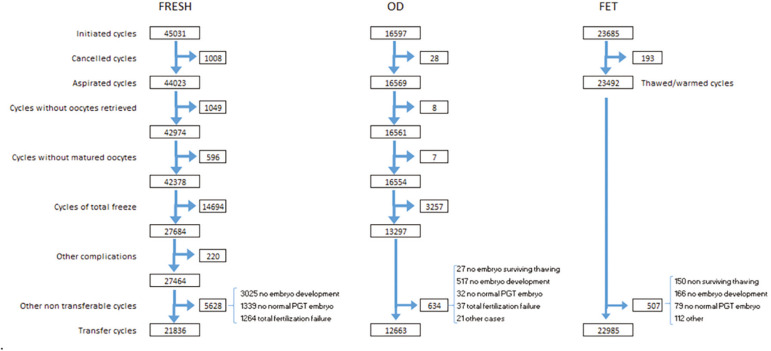
Events that affect the outcome of Fresh IVF/ICSI, Oocyte donation (OD), and Frozen/thawed embryo transfer (FET). Latin America, 2017.

Figure 1 provides a detailed description of the sequence of events that need to be considered when looking at outcome of any specific technique (IVF/ICSI, OD, FET, etc.), starting from initiated cycle, cancellations prior to follicular aspiration; aspirations with or without mature oocytes; freeze all oocytes and /or embryos; number of fertilized oocytes or failed fertilization, viability of embryos for transfer or normality of embryos after PGT. It is only after all these events have been considered and adjusted that pregnancy and delivery rates can be calculated with a well-established denominator, being initiated cycle, aspirated cycle, transfer cycle, etc. Needless to say, this detailed description is only possible in a cycle-based registry.

### ART Utilization in Latin America

**Figure 2:**
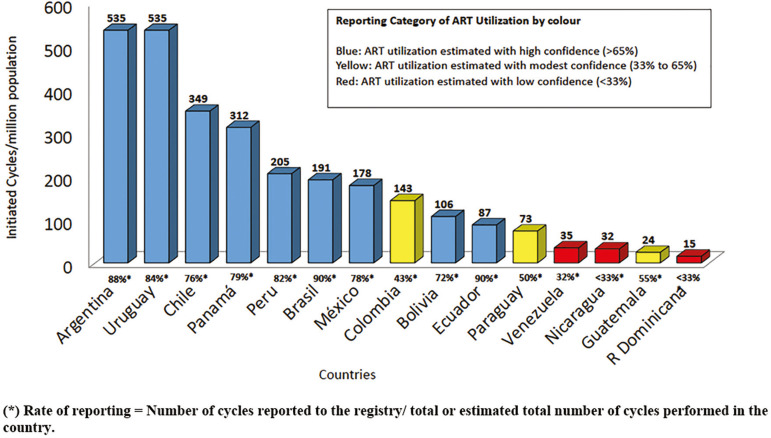
Utilization of ART. Estimated number of initiated cycles per million inhabitants by country in Latin America, 2017

Utilization of ART is expressed as the total number of cycles performed per million inhabitants (ESHRE Capri Workshop Group, 2001). Considering that not all cycles performed in every country were reported to the Latin American registry, the best possible estimate of the non-reported cycles was obtained through information provided by regional directors of REDLARA, biologists, clinicians and industry representatives. The magnitude of the estimates, which constitutes a potential source of error, was expressed as degrees of confidence according to Dyer *et al.* (2019). If the number of cycles reported to the registry exceeded 95% of the total number of cycles performed in the country, the report was considered complete. If the number of cycles reported to the registry included 64 to 94% of the total or estimated total number of cycles performed in that country, the estimate (number of cycles/million inhabitants) was considered to be of high confidence. When the proportion of cycles reported fell between 33% and 65% of the estimated total number of cycles performed in the country, results were considered of modest confidence; and if less than 33% of all cycles done in a country were reported to our registry, we assumed our numbers had low confidence. As seen in Figure 2, the RLA collects between 70 to 90% of ART cycles performed in most countries in the region, and this is specially so with the major contributors in Latin America. Overall, Argentina and Uruguay, two countries with laws providing universal care to ART have the highest utilization with 535 cycles per million followed by Chile without law but with recent public policies providing partial reimbursement with 349 cycles per million. Brazil is the major contributor in the region but its utilization is still poor.

### Age distribution

**Figure 3:**
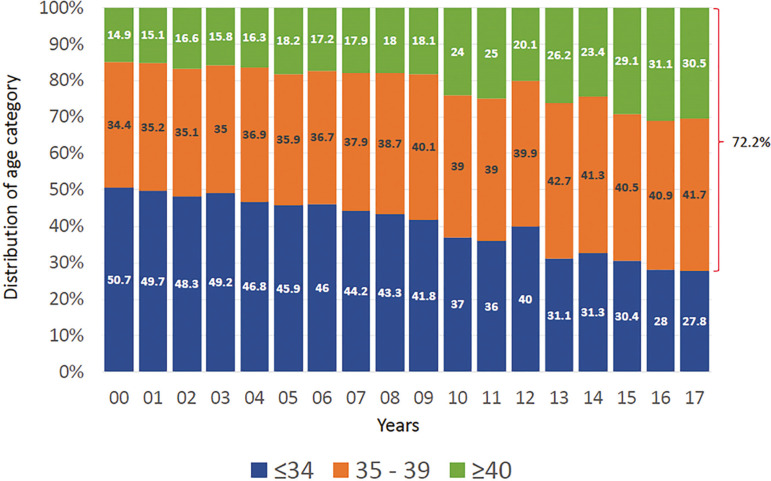
Age distribution of Female partner in IVF/ICSI. Latin America, 2000 - 2017

The mean age of women undergoing IVF/ICSI was 36.9 years (SD 4.57). The majority of cycles were performed in women aged 35 to 39 years (41.7%), followed by women aged ≥40 years (30.5%). Therefore, 72.2% of women using autologous ART were ≥35 years. In the past five years the trend is of an aging population. As seen in Figure 3, there has been a steady drop in the proportion of women aged ≤34, reaching only 27.8% in 2017 while the proportion of women ≥40 have increased from 26.2% in 2013 to 30.5% in 2017.

### Outcome of pregnancies and deliveries

#### Fresh IVF and ICSI cycles

In 2017, there were 45,031 initiated IVF/ICSI. As seen in Figure 1, after discarding aspirations without oocyte or absence of mature oocytes; and 14,694 cases of total embryo freezing; there were 27,464 oocyte retrievals exposed to the chance of achieving pregnancy and 21,836 embryo transfer cycles, generating 7,523 clinical pregnancies (CPR/OPU 27.4%).

Of these pregnancies there were 81 ectopic pregnancies (1.07%), 12 induced abortions and 1,342 miscarriages (17.8%). There were 601 losses to follow-up (7.9%) and 5,487 deliveries. Table 2 shows the clinical pregnancy rate (CPR) and delivery rate (DR) per oocyte pick-up (OPU) in IVF and ICSI cycles. ICSI represents 85.6% of fresh procedures and there were no differences in CPR or DR per oocyte retrieval between ICSI and IVF (27.3% and 27.7%. and 19.9% and 20.2%, respectively. When calculated by transfer (3359 in IVF and 18477 in ICSI), the differences in the DR per ET in IVF and ICSI, 28.3% and 25.4%, respectively, were higher than in 2016 and in the border of significance (*p*=0.0519 95% CI 0.0048% to 3.17%).

**Table 2:** Clinical pregnancy rate and delivery rate in fresh autologous IVF/ICSI cycles in 2017

Assisted reproduction technique procedure	Oocyte retrieval^a^	Clinical pregnancy rate per oocyte retrieval (%)	Delivery rate per oocyte retrieval (%)
**ICSI**	23,503	6,426 (27.3%)	4,688 (19.9%)*
**IVF**	3,961	1,097 (27.7%)	799 (20.2%)*
**TOTAL**	27,464	7,523 (27.4%)	5,487 (19.97%)
***p*-value** **b**		0.7980	0.9856

a Oocyte retrieval with at least one mature oocyte, excluding freeze-all cycles

b For IVF versus ICSI. (*) = NS

#### Oocyte donation cycles

**Figure 4:**
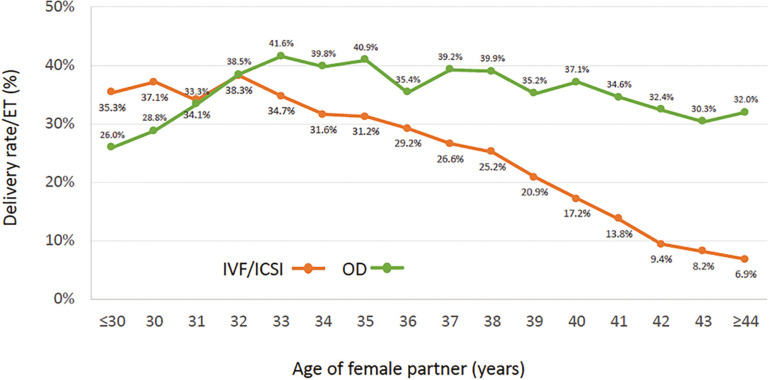
Delivery rate per embryo transfer according to age of female partner in fresh autologous IVF/ ICSI and fresh and cryopreserved oocyte donation (OD) cycles. Latin America, 2017

As seen in Figure 1, in 2017 there were 16,597 initiated cycles, and after removing freeze all cycles (oocytes and embryos) and those without suitable embryos to transfer there were 12,663 transfer cycles. As expected, both CPR and DR per ET much higher (CPR *p*<0.0001 95% CI 11.32% to 14.08%; DR *p*<0.0001 95% CI 7.01% to 9.6%) after the transfer of donated oocytes (OD) than in autologous reproduction, reaching 47.2% and 33.4%, respectively. Although CPR in Fresh/OD transfers was significantly higher than FET/OD (p=0.0001 95%CI 1.75% to 5.24%), the DR/ET was similar in both groups (Table 3). As expected, compared with autologous transfers, outcome after OD is only marginally affected by the age of the recipient (Figure 4).

**Table 3:** Clinical pregnancy rate and delivery rate by embryo transfer in oocyte donation and FET cycles in 2017

Assisted reproduction technique procedure	Embryo transfer	Clinical pregnancy per embryo transfer (%)	Delivery rate per embryo transfer (%)
**Fresh oocyte donation (OD)**	6,433	3,039 (47.2%)*	2,148 (33.4%)**
**Frozen-thawed embryo transfer (OD)**	6,230	2,725 (43.7%)*	2,116 (33.9%)**
**Frozen-thawed embryo transfer (Own)**	22,985	8,696 (37.8%)	6,539 (28.4%)

(*) *p*<0.0001;

(**) *p*=0.5643

#### Frozen embryo transfer cycles

**Figure 5:**
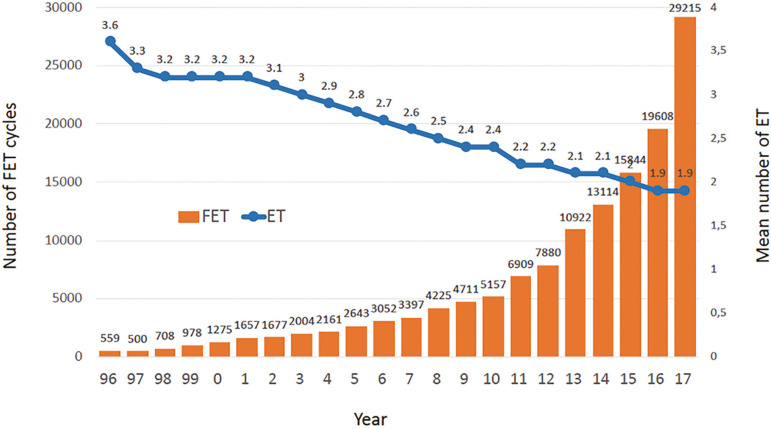
Trends in the number of frozen/thawed embryo transfer (FET) cycles and mean number of embryos transferred (ET). Latin America, 1996-2017

In 2017 there were 23,685 FET cycles representing 25.3% of all cycles performed. This represents an increment of more than 20.8% compared with 2016, while the mean number of embryos transferred has remained in 1.9, since 2016 (Figure 5). Of all initiated FET cycles, 700 cycles were discontinued. Reasons for discontinuation were non-survival and/or lack of chromosomally normal embryos (n=507; 2.1%) or abnormal endometrium (n=193; 0.8%). Therefore, out of 22,985 FET cycles, the overall CPR and DR per transfer was 37.8% and 28.4%, respectively (Table 3), which is significantly higher than 2016 (*p*=0.0006) and also, significantly higher than the CPR and DR after fresh transfers (*p*<0.0001). The higher PR and DR in FET compared with Fresh transfers are observed across all number of embryos transferred, especially in SET (Table 4 and Supplementary Table 2). 

**Table 4:** Clinical pregnancy rate, delivery rate and gestational order according to the number of embryos transferred in fresh autologous IVF/ICSI in 2017

Number of transferred embryos	Total embryo transfer		Clinical pregnancy		Deliveries							
	Number	%	Number	%	Number of deliveries	Delivery rater per embryo transfer (%)	Singleton (n)	Singleton (%)	Twin (n)	Twin (%)	≥Triplets (n)	≥Triplets (%)
1	5,881	26.9	1,481	25.2	1,070	18.2	1,044	97.6	26	2.4	0	0
2	12,798	58.6	4,902	38.3	3,615	28.3	2,845	78.7	756	20.9	14	0.4
3	2,904	13.3	1,064	36.6	749	25.8	576	76.9	159	21.2	14	1.9
≥4	253	1.2	76	30.0	53	20.9	44	83.0	7	13.2	2	3.8
Total	21,836	100	7,523	34.5	5,487	25.1	4,509	82.2	948	17.3	30	0.5

#### Number of embryos transferred, deliveries and multiple births after IVF/ICSI according to the age of women

In women ≤34 years, there were 6,309 fresh transfers. The mean number of embryos transferred was 1.87 (range 1 to 6). In this age group, 23.1% were single embryo transfers (SET), of which 45.6% were elective (eSET). Double embryo transfers (DET) corresponded to 66.8% of transfers, of which 49.6% were elective (eDET). The transfer of three embryos (TET) and 4 or more, was performed in 9.8% and 0.3% of cases.

In women aged 35 to 39 years, there were 9,692 fresh transfers. The mean number of embryos transferred was 1.89 (range 1 to 6). In this age group, 26.4% were SET, of which 28.7% were eSET. DET corresponded to 58.1% of transfers and 39.3% were eDET. The transfer of three embryos (TET) and 4 or more, were performed in 14.9% and 0.6% of cases.

In women ≥40 years of age, there were 5,835 fresh transfers. The mean number of embryos transferred was 1.89 (range 1 to 6). In this age group 31.9% were SET, of which only 12.9% were eSET, 50.9% DET, 24.8% eDET and 14.3% TET; while the transfer of four or more embryos occurred in 2.9% of transfers.

Table 4 summarizes the overall number of embryos transferred and multiple births after IVF/ICSI. The mean number of embryos transferred was 1.89 (range 1 to 6). There were 5,881 SET (26.9%), and 12,798 DET (58.6%), there were 3,157 transfers with 3 or more embryos (14.5%).

Overall, the CPR and DR per ET reached 34.5% and 25.1%, respectively. In terms of multiple births, of the 5,487 IVF/ICSI deliveries registered, 82.2% were singletons, 17.3% were twins, and 0.5% were triplets or more. Given that SET constitutes a heterogeneous group, Table 5 further stratifies IVF and ICSI outcome after transferring eSET over oSET (only 1 embryo available for transfer) and eDET over oDET (only 2 embryos available for transfer). There are huge differences both in DR/ET in both eSET and eDET over oSET and oDET; furthermore, the rate of twins and triplets increase with eDET, while eSET by itself does not seem to increase the rate of monozygotic twins.

**Table 5:** Clinical pregnancy rate, delivery rate and gestational order in elective and non-elective SET and DET in fresh autologous IVF/ICSI in 2017

Number of transferred embryos*	Total embryo transfer		Clinical pregnancy		Deliveries							
	Number	%	Number	%	Number of deliveries	Delivery rater per embryo transfer (%)**	Singleton (n)	Singleton (%)	Twin (n)	Twin (%)	≥Triplets (n)	≥Triplets (%)
oSET	4,243	72.1	789	18.6	541	12.8	528	97.6	13	2.4	0	0
eSET	1,638	27.9	692	42.2	529	32.3	516	97.5	13	2.5	0	0
oDET	7,770	60.7	2,408	31.0	1,739	22.4	1,450	83.4	284	16.3	5	0.3
eDET	5,028	39.3	2,494	49.6	1,876	37.3	1,395	74.3	472	25.2	9	0.5

* oSET or oDET: non elective single or double embryo transfer; eSET or eDET: elective single or double embryo transfer.

** DR/ET: oSET and eSET *p*<0.0001 95% CI 17.01% to 22.03%.

DR/ET: oDET and eDET *p*<0.0001 95% CI 13.26% to 16.54%.

#### Number of embryos transferred, deliveries and multiple births after OD and FET

Supplementary Table 3 summarizes the number of embryo transfers and multiple births in OD (fresh and FET), where the mean number of embryos transferred reached 1.86 (range 1 to 5). There were 3,550 SET, which correspond to 28.0% of ET and 1,012 were eSET, representing 28.5% of SET and 8% of all ET/OD. There were 7,409 DET, which correspond to 58.5% of ET, and 2,143 were eDET, representing 16.9% of all transfers in OD.

Overall, the CPR and DR per ET were 45.5% and 33.7%, respectively. Of the 4,264 deliveries registered, 75.0% were singletons, 24.2% twins and 0.8% were triplets and higher. Furthermore, DR/ET was only slightly affected by the age of the oocyte recipient (OR 0.98 95% CI 0.97-0.98) (Figure 4).

In FET cycles the mean number of embryos transferred was 1.71 (range 1 to 5). Supplementary Table 2 shows there were 8,755 SET, which correspond to 38.1%, and 12,268 DET corresponding to 53.4%. Overall, the CPR and DR per ET reached 37.8% and 28.5%, respectively. Of the 6,539 deliveries registered, 83.3% were singletons, 16.1% were twins, and 0.6% were triplets and higher.

#### Total embryo freezing

14,694 cycles of total embryo freezing were reported, 15.4% more than in 2016. On average 4.3 embryos (SD 3.1) were cryopreserved and a mean of 1.7 (1 to 4) embryos transferred. Aspirations followed by total embryo freezing gave rise to 5,856 FET cycles resulting in 1,891 deliveries and a DR/ET of 32.3%; this was higher than the DR/ET of 28.4% observed in non-freeze-all FET cycles (*p*<0.00001). A second FET attempt was reported in 1,162 cases from the same cohort, with 312 subsequent deliveries, the DR/ET in this attempt was 26.9%. Therefore, adding all transfers from this subset of total embryo freezing, the DR/ET adds to 37.3%. The mean age of women was 35.5±4.5. When stratified by number of embryos transferred, DR/ET was 29.1% in SET and 34.7% in DET.

#### Influence of stage of embryo development at transfer

Overall, 58.2% of ET were performed at the blastocyst stage, representing a 17% increment over 2016. The proportion of blastocysts transfers in FET (70.8%) almost doubled the proportion in Fresh IVF/ICSI (37.5%) This is important to consider when comparing outcome after fresh and frozen thawed transfers. In OD cycles (both fresh and frozen), the proportion of blastocyst transfers reached 69.4% which is 30% more than in 2016. Blastocyst transfer was always associated with higher DR when compared with cleavage-stage embryos, irrespective of whether fresh or frozen and the number of embryos transferred.

In IVF/ICSI, the DR after 8,185 blastocyst transfers was 31.16% compared with 21.77% after the transfer of 13,629 cleaving embryos (*p*<0.0001). In OD, the DR/ ET was 37.1% in blastocyst transfers and 25.9% in cleaving embryo (*p*<0.0001); and in FET, the proportion was 31.9% and 20.0%, respectively (*p*<0.0001).

#### Preimplantation genetic testing (PGT)

The RLA registers PGT-M and PGT-A together. 132 centers reported these procedures in 5.193 fresh cycles (11.8% of OPU); 2.353 using frozen-thawed embryos (8.1% of transfers) and 571 (8.9% of transfers) in OD. The mean age of women undergoing PGT was 38.0 (SD 4.2) among fresh cycles and 38.1 (SD 4.3) in FET.

In fresh autologous cycles, the mean number of normal embryos was 1.1 over a mean of 3.1 (SD 2.3) embryos biopsied. In FET cycles, the mean number of normal embryos was 1.9 over a mean of 3.4 biopsied. In OD, the mean number of normal embryos increased to 2.7 over a mean of 4.8 embryos biopsied. The DR/ET was 23.9% in fresh IVF/ICSI, 37.9% in FET and 43.6% in OD.

#### Miscarriage

Miscarriage rate in 7,523 pregnancies resulting from autologous fresh embryo transfer and 8,696 pregnancies of FET were 17.8% and 17.4%, respectively. As expected, miscarriage rate in a total of 3,039 OD was lower both in fresh transfers (15.6%) and in frozen/thawed OD (15.0%). Furthermore, in 672 cases of OD using FTO, miscarriage rate was also 15.0% The miscarriage rate using PGT reached 12.3% in pregnancies after FET and 14.8% in OD-FET. Table 6 provides information on the effect of PGT on miscarriage rate after FET in different age groups. When comparing miscarriage after autologous FET with and without PGT, the rate of miscarriage is significantly diminished when PGT is performed in women ≥40 from 23.7% to 9.3% (*p*<0.0001); the difference is also significant in women age 35 to 39, but in women < 35, PGT does not seem to lower the chances of miscarriage (*p*>0.3989). Furthermore, in 250 pregnancies resulting from PGT performed in FET/OD, there were 37 miscarriages (14.8%) compared with a miscarriage rate of 15.0% in FET/OD without PGT.

**Table 6:** Effect of PGT on miscarriage rate after FET in different age groups

	FET with PGT	FET without PGT	
<35	12.8%	15.3%	*p*=0.3989
35 to 39	13.9%	17.8%	*p*=0.0724
>39	9.3%	23.7%	*p*<0.0001

#### Fertility preservation (FP)

A total of 5,256 initiated cycles for FP were reported in 2017 representing 20% increase over 2016. The mean age of women was 36.1 years (≤34=27.1%, 35-39=48.7% and ≥40=24.2%). No oocytes were available for freezing in 247 follicular aspirations (4.7%). The mean number of oocytes cryopreserved was 7.5 with huge variations depending on the age of women (≤34=10.2, 35-39=7.3, and ≥40=4.9). In cases where the indication for FP was recorded, the majority were related to the desire to postpone pregnancy (3,024 cases representing 60.4%), while cancer-related factors were reported in 343 cases (6.8%); risk of premature ovarian insufficiency in 289 (5.8%) cases and other reasons in 1,353 cases (27.0%). More than 10 oocytes were cryopreserved in only 30% of women expressing the desire to postpone fertility, 39% in women having cancer and as expected, the proportion dropped to only 6% in women with risks of premature ovarian insufficiency.

### Cumulative delivery rate (CDR)

**Figure 6:**
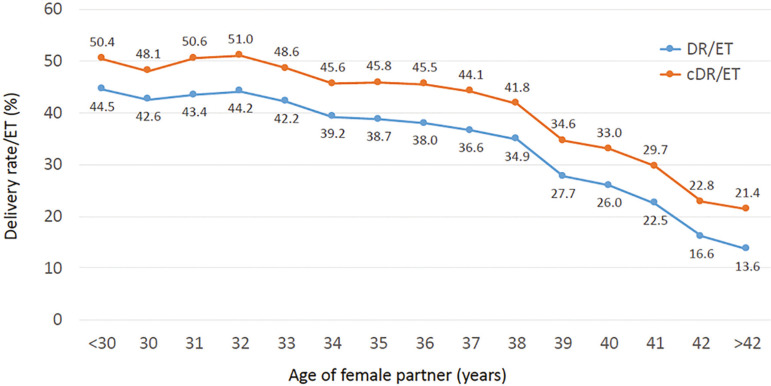
Cumulative delivery rate per embryo transfer (cDR/ET) and delivery rate per fresh embryo transfer (DR/ET) according to the age of female partner in women with surplus frozen embryos resulting from their fresh transfer. Latin America, 2017.

We were able to follow up the outcome of fresh embryo transfers and their consecutive FET in 47,492 patients between 2012 and 2017. This cohort included only women having surplus frozen embryos resulting from their fresh transfer, and only the first delivery after either fresh or frozen transfers. Taking all patients together, the DR/ET increased from 37.6% after fresh embryo transfer to a cumulative rate of 43.5% (RR 1.104; 95% CI 1.09 to 1.12; *p*<0.0001). The cumulative DR per ET stratified by the age of female partner at the time of OPU is shown in Figure 6. The increment in DR when adding FET over fresh transfers was inversely correlated to the age of the female partner. The OR for delivery was 1.3 in women <35 years (95% CI 1.2 to 1.3); 1.2 in women 35 to 39 (95% CI 1.1 to 1.3) and 1.1 in women >39 (95% CI 1.1 to 1.3).

#### Perinatal outcome and complications

Table 7 summarizes perinatal mortality. Data was available from 16,760 births and 20,093 babies born. The perinatal mortality increased from 9.4‰ births in 13,532 singletons, to 25.3‰ in 6,250 twins and 63.3‰ in 311 triplets and higher. With 1911 more babies born than in 2016, multiparity increased perinatal death in similar proportion to previous years.

**Table 7:** Perinatal mortality according to gestational order in 2017

	Singleton	Twin	≥ Triplets
Livebirth*	13,532	6,250	311
**Stillbirth**	57	63	5
**Early neonatal death**	71	99	16
Perinatal Mortality**	9.4‰	25.3‰	63.3‰

(*) Early neonatal death are excluded (**) Perinatal Mortality = (stillbirth + early neonatal death) / (livebirth + stillbirth + early neonatal death)

(**) Perinatal Mortality = (stillbirth + early neonatal death) / (livebirth + stillbirth + early neonatal death)

Gestational age at delivery was reported in 14,804 deliveries (87.2% of all deliveries). The mean gestational age at delivery was 37.7 (SD 2.3) weeks in singletons, 35.2 (SD 2.9) weeks in twins, and 31.7 (SD 2.9) weeks in triplets and higher. The overall risk of preterm birth (gestational weeks 22-36) increased from 9.5% in singletons, to 64.3% in twins, and 97.9% in triplets and higher. Furthermore, the risk of very preterm birth (gestational weeks 22-27) increased from 0.81% in singleton to 3.0% in twins and to 9.3% in triplets and higher. Table 8 shows the weight of babies born after fresh, frozen/thawed and fresh OD treatments, according to the order of gestation. As it has been reported previously (Pinborg *et al*., 2014; Schwarze *et al*., 2015), the weight of singletons born after FET (3,164±539) is significantly higher than babies born after fresh transfer (3,075±566; *p*<0.0001). A similar situation occurs after the birth of twin.

**Table 8:** Gestational age and weight at birth acccording to gestational order in 2017

ART procedure	Singleton		Twin		≥Triplets	
	Weeks of gestation	Weight grams±SD	Weeks of gestation	Weight grams±SD	Weeks of gestation	Weight grams±SD
**Fresh autologous IVF/ICSI**	37.73	3,074.5±565.5	35.06	2,246.4±568.3	31.33	1,468.1±514.2
**Autologous FET**	37.83	3,164.3±538.9	35.35	2,370.2±559.7	31.97	1,674.7±574.6
**Fresh and frozen/thawed OD**	37.36	3,008.4±582.7	35.13	2,292.2±571.6	32.07	1,641.6±464.3

## DISCUSSION

This is the 29^th^ report on ART procedures performed in Latin America. The number of new centers reporting to RLA continues to grow. Between 2016 and 2017,13 new centers were incorporated contributing to almost 5000 of the 8,126 new cycles reported in this period (8.7%). As seen in Figure 2 the majority of the 15 countries voluntarily report around 70% to 90% of the cycles performed in each country. This constitutes a noteworthy commitment of centers, which have freely kept reporting year after year for nearly 30 years. The rise in the number of initiated cycles, results in part by a 20% increment in FP, 20.8% increment in FET cycles and 15.4% rise in freeze all cycles. However, in spite of this, the drop in the proportion of twins and high order multiples has been very poor.

The mean ART utilization in 12 countries where data is reliable (Figure 2) is only 221 initiated cycles/million population which is very much under the threshold of 1,500 cycles per annum per million inhabitants proposed by the ESHRE Capri Group, in order to fulfil the needs of a population (The ESHRE Capri Workshop Group, 2001). Utilization of health services is closely associated with economic affordability. Argentina and Uruguay with laws providing universal coverage for infertility treatments have increased their utilization rate reaching 535 cycles /million inhabitants; Chile, with partial public coverage is also increasing its utilization rate but at a slower pace with only 349 cycles /million; and Brazil the largest contributor in the region, with proportionally little public support, provides less than 200 cycles/million population. This relationship confirms the importance of financial affordability in the utilization of ART. In countries with strong economic inequalities, the numbers of couples who can afford treatment are few. Public policies providing partial or complete financial support to persons requiring ART are pivotal in order to increase utilization and decrease the burden generated by infertility *per se*, as well as the burden which results from lack of access in a society with profound socio-economic disparities.

The reporting of efficacy in ART can be presented in many different ways. While there is overall agreement, that a proper outcome for ART is delivering a healthy livebirth, the main difficulty lies in what to use as denominator and how to reach international agreement in order to compare results from different latitudes. By incorporating Figure 1, we have tried to acknowledge this difficulty. If the chosen denominator is an “initiated cycle”, the freeze all cycles need to be removed because those women were not exposed to the risk of pregnancy. That accounts for 14,694 out of 45,031 fresh IVF and ICSI cycles, which leaves us with 30,337 initiated cycles where women had the real intention of becoming pregnant in that treatment. However, for the various reasons mentioned in Figure 1, only 21,836 cycles/women (72%) were in fact exposed to the chance of a livebirth after having at least one embryo transferred. This is very much dependent on the age and overall health of the population treated. In cases of young and healthy oocyte donors (OD) the proportion of dropouts due to lack of oocytes or abnormal embryos is much lower. If the freeze all cycles are removed in OD, there were 12,663 transfers which represent 94.9% of the initiated cycles exposed to the chance of pregnancy. All these clinical and biological variables need to be considered both for counseling patients and for the purpose of making results comparable.

When using large data base to provide evidence-based counselling to patients, the denominator must be initiated cycle after discarding freeze all cycles. On the other hand, if the objective is to compare the efficacy of different treatment interventions such as fresh over FET transfers or freeze all over FET that results from a failed fresh cycle, the preferred denominator is “embryos transferred”. When comparing DR after fresh transfers in Table 4 with frozen /thawed transfers in Supplementary Table 2 it is evident that FET is more efficient in both SET and DET. However, the question is whether they are comparable considering that only 37.5% of fresh transfers were blastocysts compared with 70.8% in FET. When efficacy is measured after transferring only blastocyst, the DR/ET was almost identical; 31.16% in fresh and 31.9% in FET. One can also be tempted to assume that the most efficient option is the delayed transfer after a freeze all cycle. In fact, after 5,856 FET originated from a freeze all procedure, the overall DR/ET was 32.3% compared with an overall 28.4% FET following a fresh cycle, with similar proportion of blastocysts transferred. This difference is even greater after SET (29.1% in freeze all and 25.5% in FET after a fresh cycle). Again, these two are difficult to compare. Firstly, the mean age of women during the fresh cycle that originated FET was 36.9 years while the mean age of women having freeze all was 35.5. Secondly, we must assume that in the case of a FET cycles that follows a fresh attempt, the best embryos were used in the fresh attempt and the woman was not pregnant, so the cohort of embryos left for FET from those women have a negative selection, while in the case of freeze all cycles, the best embryo is selected in the first attempt. 

What can reasonably be said is that the transfer of frozen thawed embryos does not jeopardize the chances of becoming pregnant and delivering a term livebirth as seen in Supplementary Table 2 and Table 8.

Another factor worth considering when analyzing efficacy is that providing global results for SET or DET can be misleading. As seen in Table 5 only 27.9% of SET were eSET and only 39.3% of DET were eDET. The differences in DR/ET are highly significant when the embryo selected for transfer belongs to a larger cohort of embryos available (eSET, eDET) instead of transferring one or two embryos because there were no more embryos available for transfer (oSET, oDET). The DR/ET in 1,638 eSET reached 32.3% with 2.5% of monozygotic twins. On the other hand, the transfer of eDET increased DR by only 5% over eSET, but generated 25% of twins and 0.5% triplets with severe impact in preterm births and perinatal mortality (Tables 7 and 8). 

When examining cumulative livebirths in our cohort of 47,492 patients followed between 2012 and 2017 (Figure 6). All patients were selected because they had an elective transfer during their fresh cycle, in fact, that was the reason to include them in the study of cumulative livebirths. When these curves were compared with the DR/ET in all fresh transfers in 2017 (Figure 4) the differences are huge and the results of fresh transfers in the cumulative group more closely resemble those of the fresh transfers in oocyte recipients which are mostly women with numerous embryos for transfer.

Preimplantation genetic testing (PGT) is also increasing in Latin America. Overall, 8,117 procedures were reported by 132 out of 188 institutions (ten more institutions and 2,970 more procedures than the previous year). When examining the efficacy of PGT and taking into consideration the older population treated (mean age: 38.1±4.3 years), the DR/ET, was significantly higher when comparing 1,745 cases of FET with PGT (37.9%) with 6,539 of FET without PGT (28.5%) (*p*<0.0001). The number of cases in OD and fresh transfers were too small to allow for meaningful comparisons. Furthermore, miscarriage took place in 17.6% of 16.219 pregnancies after fresh and frozen/thawed autologous cycles. This dropped to 12.8% in 845 pregnancies with PGT. In order to see the relative benefit of PGT in different age categories, miscarriage was compared with and without PGT in a standard group of only FET cycles. As seen in Table 6, there is clear benefit with PGT in women ≥40, but the benefit in younger women, although consistent, does not reach mathematical significance. The numbers are still relatively low but more and more women and men in Latin America are seeking for the illusion of certainty in delivering “normal” offspring, even in cases of OD where surprisingly 3.9% of cycles used PGT.

The concept of fertility preservation deserves special attention. When examining the data gathered in women vitrifying oocytes in order to postpone gestation, 73% were (35 (24% (40 years), and 70% of these women had less than 10 oocytes collected. This implies that a large proportion of women are living with the unrealistic expectation of becoming mothers when they so wish. The concept of fertility preservation should perhaps be replaced by oocyte preservation and at the same time, especially in emerging economies where fertility is delayed due to heavy academic or work environments, advocacy groups should contribute to educating young women and simultaneously facilitate the establishment of public policies to provide gamete preservation at younger age. 

Latin America has much room to improve. Starting with increasing access to treatment, which shall not only decrease the burden of disease, but also bridge the abysm between the rich and the poor who suffer from infertility. However, independently from many related considerations, multiple births is still a problem that needs to be dealt with on a global basis considering that of all babies born in the region, only 66.9% were singletons, while 31.4% twins, and 1.6% triplets and higher. The mean number of embryos transferred has remained quite stable in 1.9, however there are huge differences among countries in the region. While Argentina, Uruguay and Bolivia transfer a mean of 1.6 embryos, the mean number of embryos transferred in Brazil and Mexico, the two major contributors, are 2.0 and 2.2 embryos, respectively.

There is indirect evidence that when access to ART is facilitated, the number of SET cycles increase, and “success” is procured as the result of cumulative events such as fresh plus frozen transfers (Chambers *et al*., 2018). Instead, when women have only one chance in their life, the number of embryos transferred increase in order to secure pregnancy in that unique event. Nonetheless, after a critical look at these regional data, there are numerous improvements to be made in order to increase the proportion of blastocyst transfers, especially in young women, facilitating a higher proportion of eSET. Also, the good results with FET are expressed in the cumulative livebirth rates, which for young women can be as high as 50%. Any series of actions directed at facilitating access to treatment, and at the same time implementing strict quality control systems in the laboratories in order to ensure efficient long-term embryo culture and good cryopreservation techniques should be pivotal in lowering the number of embryos needed to achieve birth. The 32.3% birth rate after fresh eSET is indeed reassuring and should become a standard especially considering that when 2 embryos are electively transferred, the rise in births is only 5%, while there is a 10-fold rise in multiple births with severe impact in preterm births and perinatal mortality.

## Appendix

**Supplementary Table 1:** List of centers by country, reporting to the Latin American Registry of ART in 2017.

ARGENTINA	
•	Servicio de Medicina Reproductiva, Instituto Gamma
•	Instituto de Fertilidad Asistida
•	Centro de Estudios en Ginecología y Reproducción (CEGYR)
•	Centro de Salud Reproductiva (CER)
•	Instituto Tersoglio
•	Centro Integral de Ginecología, Obstetricia y Reproducción (CIGOR)
•	Centro de Investigaciones en Medicina Reproductiva (CIMER)
•	Centro de Medicina Reproductiva Bariloche, Fertility Patagonia
•	Centro de Estudios en Reproducción y Procedimientos de Fertilización Asistida (CRECER)
•	FECUNDITAS
•	FERTILAB
•	GESTAR
•	Centro de Reproducción Fertilequip
•	Fertya
•	Hospital de Clínicas
•	FECUNDART
•	Centro de Reproducción, servicio de Ginecología Hospital Italiano
•	Mater, Medicina Reproductiva
•	Nascentis, Medicina Reproductiva
•	HALITUS, Instituto Médico
•	Instituto Médico de Ginecología y Fertilidad PREFER
•	PREGNA, Medicina Reproductiva
•	Programa de asistencia reproductiva PROAR
•	PROCREARTE
•	Fertilidad San Isidro
•	SARESA, Salud reproductiva Salta
•	SEREMAS
•	VITAE, Medicina Reproductiva

BOLIVIA	
•	CENALFES
•	Instituto de Salud Reproductiva (ISARE)
•	EMBRIOVID, centro integral de reproducción y especialidades médicas

BRAZIL	
•	ANDROLAB, Clinica y Laboratorio de Reproducción Humana y Andrología
•	ANDROFERT, Centro de Referencia en Reproducción Masculina
•	FERTIVITRO, Centro de Reproducción Humana
•	BIOS, Centro de Medicina Reproductiva
•	FIV-MED
•	Centro de Medicina Reproductiva, Clinica Geare
•	VIDA, Centro de Fertilidad
•	Clinica FERTWAY
•	NASCER, medicina reproductiva ltda.
•	ORIGINARE, Centro de Investigación y Reproducción Humana
•	CLINIFERT, Centro de Reproducción Humana
•	CONCEPTUS, Centro de Reproducción Asistida de Ceara
•	CONCEBER, Centro de Medicina Reproductiva
•	Clinica Origen
•	Clinica Pro-Genesis
•	Centro de reproducción humana CONCEPTION
•	Centro de Reproducción Humana MONTELEONE
•	Fértile Diagnósticos
•	CEERH, Centro especializado en Reproducción Humana
•	Embrios, centro de reproducción humana
•	EMBRYOLIFE, Instituto de Medicina Reproductiva
•	Centro de Reproducción Humana, Endoscopia y Medicina Fetal de Bahía (CENAFERT)
•	Instituto VERHUM
•	Clinica FERTIBABY BH
•	Fertilcare, Medicina reproductiva
•	FECUNDA, Reproducción Humana
•	FELICCITA, Instituto de Fertilidad Ltda.
•	HUMANA, Medicina Reproductiva
•	FERTILITY, Centro de Fertilización Asistida de Campo Grande
•	FERTILITY, Centro de Fertilización Asistida
•	FERTIL Reproduccion Humana
•	REPROFERTY
•	FERTICLIN, Clínica de Fertilidad Humana
•	GENESIS, Centro de Asistencia en Reproducción Humana
•	Clinica Genics, medicina reproductiva y genómica
•	FERTIPRAXIS, Centro de Reproducción Humana
•	GERA, Grupo de endoscopia y Reproducción Asistida
•	Clinica GERAR VIDA
•	Instituto de Saude Da Mulher, Cegonha Medicina Reproductiva
•	IVI Sao Paulo, Chedid Grieco S.A.
•	HUMANA (PRIMORDIA, Medicina Reproductiva Huntington RJ)
•	Hospital de Clínicas de Riberao Preto
•	HUNTINGTON Campinas
•	HUNTINGTON, Centro de Medicina Reproductiva (Sao Paulo)
•	JULES WHITE, Centro de Medicina Reproductiva
•	HUNTINGTON Vila Mariana
•	Ideia Fertil
•	IMR, Instituto de Medicina Reproductiva e Fetal
•	Servicio de Reproducción Humana Del Hospital y Maternidad Santa Johana
•	Life reproducción humana
•	FERTILITAT, Centro de Medicina Reproductiva
•	Clínica MATRIX
•	Pro-criar Monte Sinaí
•	Centro de Reproducción Humana Nilo Frantz
•	Clínica ORIGEN
•	Procriar, Clinica de Fertilización Asistida, Blumenau
•	Clínica PRO-CRIAR, Medicina Reproductiva BH
•	Clínica PRO NASCER
•	Clinica de Reproducción PROSER SS
•	Centro de Reproducción Humana De San Jose de Rio Preto
•	GENESIS, Centro de Reproducción Humana
•	Centro de Reproducción Humana Prof. Franco Junior
•	Centro de Ensino y Pesquisa en Reproducción Asistida (Centro de Rep. Asist. Hospital Da ASA SUL)

CHILE	
•	UMR Clínica de la Mujer Antofagasta
•	Centro de Estudios Reproductivos (CER)
•	Unidad de Medicina Reproductiva, Clínica Alemana
•	Unidad de Medicina Reproductiva, Clínica las Condes
•	Unidad de Medicina Reproductiva, Clínica de la Mujer
•	UMR clínica Indisa
•	Programa e Fertilización Asistida I.D.I.M.I.
•	Clínica Monteblanco
•	Centro de Fertilidad y Medicina Reproductiva Concepción S.A.
•	Centro de reproducción humana, Valparaiso

COLOMBIA	
•	Centro FECUNDAR, Cali
•	Unidad de fertilidad del Coutry ltda. CONCEPTUM
•	Asociados en Fertilidad y Reproducción Humana
•	FERTIVIDA
•	Clinica Machicado SAS
•	Centro Médico IMBANACO
•	Instituto de Fertilidad Humana S.A.S. (INSER)
•	IN SER, Instituto Antioqueño de Reproducción
•	Procrear
•	Profamilia Fertil
•	Unidad de Fertilidad, Procreación Medicamente Asistida
•	Union temporal IN SER eje cafetero

ECUADOR	
•	Clínica de Medicina Reproductiva BIOGEPA
•	Centro Ecuatoriano de reproducción humana
•	Clínica INFES
•	Instituto Nacional de Investigación de Fertilidad y Esterilidad (INNAIFEST)
•	Instituto de Reproducción Humana Guayaquil
•	CONCEBIR, Unidad de Fertilidad y Esterilidad
•	Unidad de Fertilidad Hospital Alcívar
•	PROCREAR

GUATEMALA	
•	Centro de Reproducción Humana S.A. (CER)

MEXICO	
•	Centro de Diagnóstico Ginecológico
•	Biofertility Center
•	URA, Unidad de reproducción asistida de Hispital CIMA Hermosillo
•	Centro de Cirugía Reproductiva y Ginecología, Unidad de Fertilización In Vitro (REPROGYN)
•	Instituto de Innovación Tecnológica y Medicina Reproductiva CITMER
•	Instituto para el estudio de la Concepción Humana IECH
•	Centro de Reproducción Asistida del Hospital Español (HISPAREP)
•	Centro de Reproducción Asistida del Occidente
•	Centro de Reproducción Asistida de Saltillo
•	Centro Universitario de Medicina Reproductiva
•	Fertility Center Cancún
•	Genesis Centro de Fertilidad (Culiacan)
•	Ginecología y Reproducción Asistida GYRA
•	Grupo de reproducción y genética AGN y asociados
•	Instituto para el estudio de la concepción humana de Baja California
•	Instituto Mexicano de Alta Tecnología Reproductiva S.C. (INMATER)
•	Instituto de medicina reproductiva del Bajío IMER, sede Guadalajara
•	Instituto IMER de Tijuana
•	Instituto Médico de la mujer (RED CREA)
•	Instituto de Ciencias en Reproducción Humana, sede Guadalajara
•	Instituto de Ciencias en Reproducción Humana, sede Matamoros
•	Centro especializado para la atención de la mujer (CEPAM)
•	INGENES DF
•	INGENES Guadalajara
•	Ingenes Monterrey
•	Instituto de Ciencias en Reproducción Humana (VIDA), sede León
•	Medica Fertil, Querétaro
•	Instituto de ciencias en reproducción humana del Sureste (Vida Merida)
•	Clinica Nascere
•	Centro de Medicina Reproductiva FILIUS
•	Plenus, Reproducción Asistida
•	PROGEN, Reproducción asistida y medicina fetal
•	Centro de Reproducción Asistida Santa Fe SA de CV (PRONATAL)
•	Clinica de Infertilidad y reproducción asistida de Toluca SA de CV
•	Centro especializado en esterilidad y Reproducción Humana (CEERH)
•	Instituto de Ciencias en reproducción humana VIDA, ciudad de Mexico.
•	VILTIS
•	Vida, Instituto de Reproducción Humana del Noroeste, Tijuana

NICARAGUA	
•	Centro de Fertilidad de Nicaragua

PANAMA	
•	IVI Panamá S.A.
•	Centro de reproducción Punta Pacífica
•	Instituto de salud femenina

PARAGUAY	
•	Neolife, Medicina y cirugía reproductiva

PERU	
•	Clínica CEFRA, Centro de Fertilidad y Reproducción Asistida
•	CERFEGIN
•	Centro de Fertilidad y Ginecología del Sur (CFGS)
•	Clinica de fertilidad del norte, Clinifer de Chiclayo
•	FERTILAB, Laboratorio de Reproducción asistida
•	Inmater, Clinica de fertilidad
•	Instituto de Reproducción de la Clinica Ricardo Palma
•	Clinica Miraflores
•	Nacer
•	Grupo Pranor, Clínica CONCEBIR
•	Grupo Pranor, Instituto de Ginecología y Reproducción
•	Pranor, laboratorio de medicina reproductiva sede trujillo

REPUBLICA DOMINICANA	
•	Instituto de reproducción y ginecología del Cibao (IREGCI)
•	PROFERT

URUGUAY	
•	Centro de Esterilidad Montevideo (CEM)
•	Centro de Reproducción Humana del Interior

VENEZUELA	
•	FERTILAB
•	EMBRIOS, Centro de Fertilidad y Reproducción Humana, Hospital de Clínicas de Caracas
•	Instituto Venezolano de Fertilidad
•	Laboratorios In Vitro de Venezuela

**Supplementary Table 2:** Clinical pregnancy rate, delivery rate and gestational order according to the number of embryos transferred in FET in 2017.

Number of transferred embryos	Total embryo transfer		Clinical pregnancy		Deliveries							
	Number	%	Number	%	Number of deliveries	Delivery rater per embryo transfer (%)	Singleton (n)	Singleton (%)	Twin (n)	Twin (%)	≥Triplets (n)	≥Triplets (%)
1	8,755	38.1	3,000	34.3	2,229	25.5	2,195	98.5	34	1.5	0	0.0
2	12,268	53.4	4,968	40.5	3,775	30.8	2,883	76.4	873	23.1	19	0.5
3	1,872	8.1	702	37.5	518	27.7	361	69.7	138	26.6	19	3.7
≥4	90	0.4	26	28.9	17	18.9	11	64.7	5	29.4	1	5.9
Total	22,985	100.0	8,696	37.8	6,539	28.4	5,450	83.3	1050	16.1	39	0.6

**Supplementary Table 3:** Clinical pregnancy rate, delivery rate and gestational order according to the number of embryos transferred in fresh and frozen oocyte donation cycles in 2017.

Number of transferred embryos	Total embryo transfer		Clinical pregnancy		Deliveries							
	Number	%	Number	%	Number of deliveries	Delivery rater per embryo transfer (%)	Singleton (n)	Singleton (%)	Twin (n)	Twin (%)	≥Triplets (n)	≥Triplets (%)
1	3,550	28.0	1,427	40.2	1,033	29.1	1,013	98.1	19	1.8	1	0.1
2	7,409	58.5	3,499	47.2	2,585	34.9	1,803	69.7	772	29.9	10	0.4
3	1,674	13.2	825	49.3	637	38.1	376	59.0	239	37.5	22	3.5
≥4	30	0.3	13	43.3	9	30.0	7	77.8	2	22.2	0	0.0
Total	12,663	100.0	5,764	45.5	4,264	33.7	3,199	75.0	1,032	24.2	33	0.8
